# Orthogonal Optimization, Characterization, and In Vitro Anticancer Activity Evaluation of a Hydrogen Peroxide-Responsive and Oxygen-Reserving Nanoemulsion for Hypoxic Tumor Photodynamic Therapy

**DOI:** 10.3390/cancers15051576

**Published:** 2023-03-03

**Authors:** Liang Hong, Jianman Wang, Yi Zhou, Guofu Shang, Tao Guo, Hailong Tang, Jiangmin Li, Yali Luo, Xiangyu Zeng, Zhu Zeng, Zuquan Hu

**Affiliations:** 1 Key Laboratory of Infectious Immune and Antibody Engineering in University of Guizhou Province, Engineering Research Center of Cellular Immunotherapy of Guizhou Province, School of Basic Medical Sciences/School of Biology and Engineering (School of Modern Industry for Health and Medicine), Guizhou Medical University, Guiyang 550025, China; 2 Immune Cells and Antibody Engineering Research Center in University of Guizhou Province, Key Laboratory of Biology and Medical Engineering, Guizhou Medical University, Guiyang 550025, China; 3 Key Laboratory of Environmental Pollution Monitoring and Disease Control, Ministry of Education of China, Guizhou Medical University, Guiyang 550025, China

**Keywords:** hypoxia, nanoemulsion, photodynamic therapy, tumor

## Abstract

**Simple Summary:**

Tumor hypoxia can significantly reduce the effectiveness of photodynamic therapy (PDT). One approach to addressing this issue is in situ oxygen generation, which involves using catalysts such as catalase to decompose the excess H_2_O_2_ produced by tumors. While this strategy can be specific to tumors, its effectiveness is limited by the usually low tumor H_2_O_2_ levels. Another approach, oxygen delivery, involves using substances with high oxygen solubility, such as perfluorocarbon, to transport oxygen for use in PDT. While this method can be effective, it lacks specificity for tumors. To combine the benefits of both approaches, we developed a nanoemulsion system CCIPN. The perfluoropolyether in CCIPN could store oxygen generated by catalase within the same nanoplatform for use in PDT. CCIPN was created using an optimized sonication-phase inversion composition–sonication method, and its properties and anticancer activity were studied in vitro. This research contributes to the design and production of oxygen-supplementing PDT nanomaterials.

**Abstract:**

Tumor hypoxia can seriously impede the effectiveness of photodynamic therapy (PDT). To address this issue, two approaches, termed in situ oxygen generation and oxygen delivery, were developed. The in situ oxygen generation method uses catalysts such as catalase to decompose excess H_2_O_2_ produced by tumors. It offers specificity for tumors, but its effectiveness is limited by the low H_2_O_2_ concentration often present in tumors. The oxygen delivery strategy relies on the high oxygen solubility of perfluorocarbon, etc., to transport oxygen. It is effective, but lacks tumor specificity. In an effort to integrate the merits of the two approaches, we designed a multifunctional nanoemulsion system named CCIPN and prepared it using a sonication-phase inversion composition–sonication method with orthogonal optimization. CCIPN included catalase, the methyl ester of 2-cyano-3,12-dioxooleana-1,9(11)-dien-28-oic acid (CDDO-Me), photosensitizer IR780, and perfluoropolyether. Perfluoropolyether may reserve the oxygen generated by catalase within the same nanoformulation for PDT. CCIPN contained spherical droplets below 100 nm and showed reasonable cytocompatibility. It presented a stronger ability to generate cytotoxic reactive oxygen species and consequently destroy tumor cells upon light irradiation, in comparison with its counterpart without catalase or perfluoropolyether. This study contributes to the design and preparation of oxygen-supplementing PDT nanomaterials.

## 1. Introduction

Photodynamic therapy (PDT) has been recognized as a promising modality to treat tumors. Typically, PDT utilizes the toxic reactive oxygen species (ROS) converted from the oxygen molecules surrounding the photosensitizers under light irradiation to kill cancer cells [1,2,3]. Owing to the unique characteristics of light, PDT has a high tumor-targeting ability, low side effects, and low invasiveness, which are favorable properties for tumor treatments [4,5,6]. Photosensitizers can be innovated to obtain functions, such as targeting oncogenic mutations [7], targeting epigenetic alterations [8], and targeting tumors with molecules that are required for cancer growth [9], which can improve the anti-cancer effect. Despite their valuable properties, the efficiency of photosensitizers in converting oxygen to cytotoxic ROS is seriously limited by the hypoxic state during the PDT treatment process. On the one hand, the tumor microenvironment is innately hypoxic due to the rapid tumor cell proliferation and abnormal tumor blood vessels [10,11]. On the other hand, the PDT reaction consumes oxygen, which exacerbates hypoxia [12]. Therefore, it is critical to overcome hypoxia for the purpose of improving PDT efficiency. Moreover, it has also been recognized that hypoxia promotes tumor metastasis and resistance to treatment methods including chemotherapy, radiotherapy, and immunotherapy [13,14]. Therefore, alleviation of the hypoxic state might also improve the therapeutic effect of these therapies if they are combined with PDT [15].

To address hypoxia in PDT, many strategies based on innovative material preparation have been developed. The oxygen-supplementing strategy has attracted wide attention owing to its ability to alter the hypoxic tumor microenvironment and, thereby, its potential to inhibit tumor metastasis. The oxygen-supplementing strategy can be categorized briefly into two methods: in situ oxygen generation and oxygen delivery. The in situ oxygen generation method uses catalase or metal-based catalysts to accelerate the decomposition of tumor-overproduced hydrogen peroxide (H_2_O_2_) into oxygen [16,17]. The other strategy, oxygen delivery, relies on materials having high oxygen solubility, such as perfluorocarbon and hemoglobin, to transport oxygen and gather oxygen around photosensitizer molecules [2,18,19]. Perfluorocarbons have intrinsically higher oxygen loading efficiency than hemoglobin-based formulations. Under 1 atm at 25 °C, 40–50 mL oxygen can be loaded by 100 mL of perfluorocarbon, while only ~20 mL oxygen could be solubilized by 100 mL of blood (hemoglobin concentration 150 g L^−1^, pO_2_ ~159 mmHg) [20,21]. The in situ oxygen generation strategy and oxygen delivery strategy have their own strengths and shortcomings. The in situ oxygen generation strategy offers high tumor specificity because the level of H_2_O_2_ in the tumor site is higher than that in normal tissue. However, the tumor H_2_O_2_ level (<100 µM) is still too low to generate adequate oxygen for PDT [22]. In contrast, the oxygen delivery strategy possesses no intrinsic tumor specificity, but can supply plenty of oxygen molecules and be used as an efficient oxygen reservoir.

In this context, herein we designed and prepared a multifunctional nanoemulsion system, aiming at integrating the merits of the in situ oxygen generation strategy and oxygen delivery strategy. This multifunctional nanoemulsion system contains catalase, a synthetic triterpenoid the methyl ester of 2-cyano-3,12-dioxooleana-1,9(11)-dien-28-oic acid (CDDO-Me), photosensitizer IR780, and perfluoropolyether. It is named the catalase-CDDO-Me-IR780-perfluoropolyether nanoemulsion, and abbreviated as CCIPN. When delivered to tumor cells via an enhanced permeability and retention (EPR) effect and/or nanomaterial-induced endothelial leakiness (NanoEL) effect [23], CCIPN could achieve a cascade effect. The catalase decomposes the tumor-overproduced H_2_O_2_ into oxygen, and the oxygen is then reserved in the perfluoropolyether for PDT. CCIPN combines the tumor specificity of the in situ oxygen generation strategy and the high efficiency of the oxygen delivery strategy. Moreover, CDDO-Me has been reported to remodel the immunosuppressive tumor microenvironment and enhance the therapeutic efficacy of immunotherapy [24]. Therefore, the CDDO-Me loaded within CCIPN might boost the antitumor immune response of the body, and provides CCIPN with the potential to be synergized with immunotherapy. To prepare CCIPN, we used a sonication-phase inversion composition–sonication (S-PIC-S) method that we developed by advancing our previous sonication and phase-inversion composition (SPIC) method [25]. The S-PIC-S method could further reduce the production droplet size while retaining the advantage of environment-sensitive substance (herein, IR780) protection [26]. The preparation process of the S-PIC-S method was optimized by orthogonal tests in terms of system compositions and operation parameters. Afterwards, the properties of CCIPN were investigated. In addition, the cytocompatibility and the ability of CCIPN to generate ROS and kill cancer cells were evaluated in vitro. This research provides insights into the rational design and convenient preparation of oxygen-supplementing nanoemulsion systems for PDT and possible combined PDT and immunotherapy.

## 2. Materials and Methods

### 2.1. Materials

IR780 iodide (purity ≥ 95.0%) was purchased from Bellingway Technology Co., Ltd. (Beijing, China). Bardoxolone methyl (CDDO-Me) and perfluoropolyether with molecular weight 6000 (PFPE MW 6000) were purchased from Maclean Biochemical Technology Co., Ltd. (Shanghai, China). Catalase (CAT, 2000–5000 units/mg protein) from bovine liver was obtained from Sigma-Aldrich^®^ (Shanghai, China). Minimum essential medium (MEM), Ham’s F-12K medium, Dulbecco’s Modified Eagle Medium (DMEM), fetal bovine serum (FBS), and Tween 20 and acetonitrile (purity ≥ 99.9%) were purchased from Chaoyuan Zhicheng Biotechnologies Co., Ltd. (Guiyang, China). Fomblin^®^ Y (MW 1800), 1H-tridecafluorohexane (MW 320.05), perfluorooctane (MW 438.06), and 1-bromoheptadecafluorooctane (MW 498.96) were purchased from Aladdin Biochemical Technology Co., Ltd. (Shanghai, China). Syringe filters with a pore size of 0.22 μm and 0.45 μm (PTFE hydrophilic) were purchased from Navigator Lab Instrument Co., Ltd. (Tianjin, China). Deionized water was produced by ELGA VEOLIA (Veolia Water Solutions & Technologies, Shanghai, China).

### 2.2. Selection of Perfluorocarbon Type for CCIPN

To investigate the influence of the perfluorocarbon type on the droplet diameter and IR780 EE, five diverse perfluorocarbons (Fomblin^®^ Y, 1H-tridecafluorohexane, perfluorooctane, 1-bromoheptadecafluorooctane, and PFPE MW 6000) were used to produce nanoemulsions with similar total compositions (3.6 mL PBS containing 16 mg CAT, 0.28 g surfactant [Tween 20], 0.12 g perfluorocarbon, 4 mg IR780, and 2 mg CDDO-Me) using the SPIC method. Primarily, a multipoint magnetic stirrer (CJB-S-10D, Yuming Instrument equipment Co., Ltd., Shanghai, China) was used to stir the aforementioned mixture for 10 min (750 r min^−1^). The bottle containing the mixture was then partly submerged in water and bath-sonicated for 20 min utilizing ultrasonic cleaning equipment (SB-4200D, Xinzhi Biotechnology Co., Ltd., Ningbo, China). Afterwards, a multipoint magnetic stirrer was employed to stir the mixture in the bottle for 20 min (750 r min^−1^). The water phase (3.6 mL PBS, pH 7.4 containing 16 mg CAT) was then titrated into the mixture with a peristaltic pump (BT100J-1A, Huiyu Weiye Fluid Equipment Co., Ltd., Beijing, China) when the mixture was continuously agitated at 750 r min^−1^. The rotation speed of the peristaltic pump was set as 22.0 rpm, and the titration rate was approximately one drop every 3 s. When the process of adding the water phase was finished, agitation proceeded for 30 min. The product was then centrifuged for 3 min (3000 r min^−1^) using a centrifuge (H1650-W, Xiangyi Centrifuge Instrument Co., Ltd., Changsha, China). Finally, 3 mL supernatant was taken and filtered with firstly 0.45 μm and then 0.22 μm syringe filters. A UV–visible spectrophotometer (A360, Aoyi Instruments Co., Ltd., Shanghai, China) was utilized to measure the absorbance at 793 nm (i.e., absorption peak value of IR780 emulsions). A Brookhaven 90 Plus PALS dynamic light scattering (DLS) machine (Brookhaven Instruments Corporation, Holtsville, NY, USA) was used to detect the mean droplet size at room temperature. A standard curve for the relationship between the IR780 concentration and absorbance at 793 nm was established and used to calculate the IR780 concentration and subsequently the IR780 EE. An acetonitrile solution of 2 mg mL^−1^ IR780 was diluted for the standard curve by preparing a gradient of IR780 concentration with 0.0013, 0.002, 0.0027, 0.0033, and 0.004 mg mL^−1^. The IR780 EE of CCIPN can be calculated from Equation (1):

(1) $$EE\left(\%\right)=100\%\times C_{t}V/m$$

where C_t_ is the tested IR780 concentration, V is the volume of the system (in Section 2.2, 4 mL and in other sections, 10 mL), and *m* is the weight of initially used IR780.

### 2.3. Determination of Added CDDO-Me Amount and CAT Distribution Ratio for CCIPN

Unless otherwise stated, the emulsion was prepared in the following way using the SPIC method. First, a multipoint magnetic stirrer was used to stir the organic phase consisting of Fomblin^®^ Y (0.3 g), Tween 20 (0.7 g), IR780 (10 mg), and CDDO-Me (5 mg) for 10 min (750 r min^−1^). The bottle containing the organic phase was then partly submerged in water and sonicated for 20 min utilizing ultrasonic cleaning equipment (SB-4200D, Xinzhi Biotechnology Co., Ltd., Ningbo, China). Afterwards, the multipoint magnetic stirrer was used to stir the organic phase for 20 min (750 r min^−1^). After this, the water phase (9 mL PBS, pH 7.4 containing 40 mg CAT) was titrated into the organic phase at ~1 drop every 3 s (pump rotation speed 22.0 rpm) with a ristaltic pump (BT100J-1A, Huiyu Weiye Fluid Equipment Co., Ltd., Beijing, China) while the organic phase was under continual agitation (750 r min^−1^). When the process of adding the water phase was finished, agitation was continued for 30 min. Then, the emulsion was centrifuged for 3 min (3000 r min^−1^) using a centrifuge (H1650-W, Xiangyi Centrifuge Instrument Co., Ltd., Changsha, China). Finally, 3 mL supernatant was taken and filtered using firstly 0.45 μm and then 0.22 μm syringe filters. The production process was conducted at 25 °C. In some assays, certain parameters were changed to explore their influence on the size of the droplet and IR780 EE prepared by the abovementioned method.

#### 2.3.1. Effect of Added CDDO-Me Amount on Droplet Size and IR780 EE

The influence of the added CDDO-Me amount on the droplet diameter and IR780 EE of CCIPN was explored by varying the amount (0, 1, 2, or 5 mg) of CDDO-Me added to the 10 mL system.

#### 2.3.2. Effect of CAT Distribution on Droplet Size and IR780 EE

The effect of CAT distribution on the droplet diameter and IR780 EE was examined by altering the organic-phase-to-aqueous-phase ratio of CAT (0, 1:3 or 1:1) when the total CAT amount was set as 40 mg. The diverse organic-phase-to-aqueous-phase ratios were achieved by adding a certain amount of CAT to the organic phase in the beginning of the preparation process. 

### 2.4. Orthogonal Optimization for Preparation of CCIPN Using S-PIC-S Method

During all the orthogonal assays, the weight of CDDO-Me added was 1 mg, and the organic-phase-to-aqueous-phase ratio of CAT was 1:3. The CCIPNs were prepared using the S-PIC-S method as follows. First, a multipoint magnetic stirrer was used to agitate the organic phase consisting of Fomblin^®^ Y (0.3 g), Tween 20 (0.7 g), IR780 (10 mg), CDDO-Me (1 mg) and CAT (certain amount) for 10 min (750 r min^−1^) at 25 °C. The bottle containing the organic phase was then partly submerged in water and bath-sonicated for 20 min utilizing ultrasonic cleaning equipment (SB-4200D, Xinzhi Biotechnology Co., Ltd., Ningbo, China). Afterwards, the multipoint magnetic stirrer was used to stir the organic phase for 20 min (750 r min^−1^). Then, the water phase (9 mL PBS, pH 7.4 containing a certain amount of CAT) was added into the organic phase at ~1 drop every 3 seconds (pump rotation speed 22.0 rpm) with a peristaltic pump (BT100J-1A, Huiyu Weiye Fluid Equipment Co., Ltd., Beijing, China) when the organic phase was under continuous agitation (750 r min^−1^). After the water phase’s addition, agitation was continued for 30 min. Then, the system was probe sonicated using an Ultrasonic Homogenizer (JY92-ⅡN, Xinzhi Biotechnology Co., Ltd., Ningbo, China). Subsequently, the system was centrifuged for 3 min (3000 r min^−1^) using a centrifuge (H1650-W, Xiangyi Centrifuge Instrument Co., Ltd., Hunan, China). Finally, 4 mL supernatant was taken and filtered using the 0.45 μm syringe filter. 

The influences of the total CAT concentration (including the CAT in both the aqueous phase and organic phase, 2, 4 and 6 mg mL^−1^), ultrasonic power (50%, 60% and 70%), ultrasonic time (30, 60 and 90 s), and stirring temperature during the first 10 min (25, 30 and 35 °C) on the droplet size and IR780 EE were examined. The levels and factors of the orthogonal experiment are shown in Table 1. In this research, because there were three levels and four factors, the L_9_(3^4^) orthogonal experiment was used. The mean droplet diameters of the CCIPNs were measured using the Brookhaven 90 Plus PALS equipment at 25 °C. The IR780 EE was calculated from the aforementioned Equation (1).

### 2.5. The Optimized CCIPN Preparation

First, a multipoint magnetic stirrer was used to stir a bottle of organic phase consisting of Fomblin^®^ Y (0.3 g), Tween 20 (0.7 g), IR780 (10 mg), CDDO-Me (1 mg) and CAT (5 mg) for 10 min (750 r min^−1^) at 25 °C. The bottle was then partly submerged in water and bath-sonicated for 20 min utilizing ultrasonic cleaning equipment (SB-4200D, Xinzhi Biotechnology Co., Ltd., Ningbo, China). After, the multipoint magnetic stirrer was used to agitate the organic phase for 20 min (750 r min^−1^). The water phase (9 mL PBS, pH 7.4 containing 15 mg CAT) was then added into the organic phase at ~1 drop every 3 seconds (pump rotation speed 22.0 rpm) with a peristaltic pump (BT100J-1A, Huiyu Weiye Fluid Equipment Co., Ltd., Beijing, China) when the organic phase was under continual agitation (750 r min^−1^). After the process of adding the water phase was finished, agitation was continued for 30 min. The system was then probe sonicated using an Ultrasonic Homogenizer (JY92-ⅡN, Xinzhi Biotechnology Co., Ltd., Ningbo, China). Afterwards, the emulsion was centrifuged for 3 min (3000 r min^−1^) using a centrifuge (H1650-W, Xiangyi Centrifuge Instrument Co., Ltd., Changsha, China). Finally, 4 mL supernatant was taken and filtered using the 0.45 μm syringe filter. The production process was conducted at 25 °C.

### 2.6. Droplet Size Measurements, UV–Visible Light Absorption Spectrum and Fluorescence Spectrum

A Brookhaven 90 Plus PALS instrument was employed to detect the mean droplet diameters and polydispersity index (PDI) of the optimized CCIPNs at 25 °C. Here, 1.33 was set as the refractive index. All samples were diluted 60 times with deionized water prior to measurements. When the preparation of CCIPNs was finished, the mean droplet diameter and PDIs were determined directly. The blank nanoemulsions utilized in the UV–visible light absorption spectra detection were produced similarly to the CCIPNs, except that no IR780 was added. A UV–visible spectrophotometer was employed to record the absorption spectra at 25 °C. Fluorescence spectra were measured using cuvettes with a 1 cm path length and a slit width of 10 nm at 298 K on a Fluorescence-4600 spectrometer (Hitachi High-Technologies Corporation, Tokyo, Japan).

### 2.7. Transmission Electron Microscopy (TEM) and Scanning Electron Microscopy (SEM)

TEM and SEM were used to record the morphology of the CCIPN droplets. To accomplish this, 10 μL CCIPN was dropped onto a carbon-coated copper grid, dried naturally, and photographed using TEM (JEOL-2100F, JEOL Ltd., Tokyo, Japan). 10 μL CCIPN was diluted 100 times and 10 μL samples of the diluted emulsions were dropped onto a carbon-coated copper grid, and photographed using SEM (Phenom XL etc., FEI Electron Optics BV, Eindhoven, The Netherlands) after the sample had dried naturally.

### 2.8. Physical and Chemical Stability of CCIPN

The physical and chemical stability of CCIPN in the physiological environment and during storage at 4 °C were investigated. The alteration of the droplet size measured by DLS was used to evaluate the physical stability, and the variations in the UV–visible light absorption were applied to examine the chemical stability. To study their stability in the physiological condition, the CCIPNs were diluted at a ratio of 1:60 with PBS (pH 7.4) and stored in incubators at 37 °C (GNP-9080BS-Ⅲ, Xinmiao Medical Device Manufacturing Co., Ltd., Shanghai, China). The droplet size and absorbance at 793 nm were recorded after storage for 0, 12, 24, 36, or 48 h. For the storage stability of CCIPN at 4 °C, droplet size and UV–visible light absorption spectrum detections were conducted every 3 days during the 15-day storage period. All of the emulsions were diluted 50 times with deionized water and then determined.

### 2.9. Cell Culture

Human PC-3 prostate cancer cells and DU145 prostate cancer cells were obtained from Procell Life Science & Technology Co., Ltd. (Wuhan, China). Human Umbilical Vein Endothelial Cells (HUVECs) were obtained from Shunran Biotechnology Company (Shanghai, China). The PC-3 cells, DU145 cells and HUVECs were cultured in Ham’s F-12K medium, MEM and DMEM, respectively. Each type of medium contained 10% FBS and 1% penicillin–streptomycin solution. The cells were cultivated at 37 °C in a humidified incubator with 5% CO_2_ and ~21% O_2_.

### 2.10. Cytocompatibility Assays

For cytocompatibility assays, 1.0 × 10^4^ HUVECs or DU145 cells were placed into each well of the 96-well plates. The plates were incubated for 24 h in a 37 °C incubator with 5% CO_2_. The complete medium was then substituted by DMEM (for HUVECs) or MEM (for DU145 cells) containing diverse concentrations of CCIPNs (0, 0.025, 0.05 and 0.075 µg mL^−1^, IR780 concentrations). After incubation at 37 °C with 5% CO_2_ for 24 h, the medium was replaced with 100 µL of PBS containing 10 µL CCK-8. Afterwards, the cells were incubated for 1.5 h at 37 °C. Lastly, a Microplate Reader (ELX800, Bio Tek instruments, Inc., Highland Park, USA) was employed to measure the absorbance at 450 nm. In addition, the morphologies of cells incubated for 24 h with the CCIPNs were recorded utilizing a CYTATION/5 imaging reader (Bio Tek instruments, Inc., Winooski, VT, USA).

### 2.11. Phototoxicity Assays

The phototoxicity assays were conducted on a cancerous cell line (DU145 prostate cancer cells) and a normal cell line (HUVECs) under hyperoxic condition (~21% O_2_), and on a cancerous cell line (PC-3 prostate cancer cells) under hypoxic condition (1% O_2_). For comparison, CINs were prepared similarly to the CCIPNs but without CAT and perfluoropolyether involvement.

The phototoxicity assays on DU145 cells under hyperoxic condition (~21% O_2_) were performed as follows: DU145 cells were placed into two separate 96-well plates at 1.2 × 10^4^ cells well^-1^ and incubated for 24 h in a 37 °C incubator with 5% CO_2_. The complete medium was then displaced by CCIPNs or CINs, which were diluted with MEM to 0.075 µg mL^−1^ (IR780 concentration). In addition, MEM was used to treat the cells and served as a control. After incubation at 37 °C and 5% CO_2_ for 3 h, the medium was replaced with 100 µL MEM. Subsequently, the cells in one plate were irradiated with a laser device (MDL-Ⅲ-785 nm-2.5 W-CJ11170, New Industries Optoelectronics Tech. Co., Ltd., Changchun, China) for 5 min, and the cells in the other plate received no irradiation. The distance between the laser device and the plate, the optical power density and the wavelength of the laser were 20 cm, 0.579 W/cm^2^ and 785 nm, respectively. The cells were then incubated at 37 °C and 5% CO_2_ for 12 h. Afterwards, the liquids were substituted by PBS containing 10% CCK-8. After incubation at 37 ◦C and 5% CO_2_ for 3 h, a Microplate Reader (ELX800, Bio Tek instruments, Inc., Highland Park, IL, USA) was used to measure the absorbance at 450 nm. The cell morphologies were recorded utilizing a CYTATION/5 imaging reader (Bio Tek instruments, Inc., Winooski, VT, USA).

The phototoxicity tests on HUVECs under hyperoxic condition (~21% O_2_) were conducted as follows: HUVECs were planted into a 96-well plate at 1.2 × 10^4^ cells well^−1^ and incubated for 24 h at 37 °C and 5% CO_2_. Afterwards, the complete medium was substituted with CCIPNs or CINs, which were diluted with DMEM to 0.075 µg mL^−1^ (IR780 concentration). Meanwhile, DMEM was used to treat cells and served as a control. After incubation at 37 °C and 5% CO_2_ for 3 h, the liquids were substituted with DMEM. Then, the cells were treated with or without laser irradiation. The laser was generated by a laser device (MDL-Ⅲ-785 nm-2.5 W-CJ11170, New Industries Optoelectronics Tech. Co., Ltd., Changchun, China). The distance between the laser device and the plate, the optical power density, the wavelength of the laser, and the irradiation time were 20 cm, 0.579 W/cm^2^, 785 nm, and 5 min, respectively. Afterwards, the cells were incubated at 37 °C and 5% CO_2_ for 12 h. The medium was then replaced with PBS containing 10% CCK-8. Lastly, the cells were incubated at 37 °C for 3 h and the absorbance at 450 nm was measured by a Microplate Reader (ELX800, Bio Tek instruments, Inc., Highland Park, IL, USA). The cell morphologies were photographed using a CYTATION/5 imaging reader (Bio Tek instruments, Inc., Winooski, VT, USA).

The phototoxicity tests on PC-3 cells under hypoxic condition (1% O_2_) were performed as follows: PC-3 cells were placed into 96-well plates at 1.2 × 10^4^ cells well^−1^ and incubated for 12 h in a 37 °C incubator with 5% CO_2_. The complete medium was then displaced by CCIPNs or CINs, which were diluted with MEM to 0.075 µg mL^−1^ (IR780 concentration). In addition, Ham’s F-12K medium was used to treat the cells and served as a control. The hypoxic condition was created by setting the gas composition to 1% O_2_ on an incubator [HF100 (Tri-Gas), Lishen Scientific Instrument Co., Ltd., Shanghai, China]. After incubation at 37 °C, 1% O_2_ and 5% CO_2_ for 1 h, the liquid was replaced by Ham’s F-12K medium, and the incubation continued for 30 min at 37 °C, 1% O_2_ and 5% CO_2_. Subsequently, the cells were irradiated with a laser device (MDL-Ⅲ-785 nm-2.5 W-CJ11170, New Industries Optoelectronics Tech. Co., Ltd., Changchun, China) for 6 min, or received no irradiation. The distance between the laser device and the plate, optical power density, and wavelength of the laser were 20 cm, 0.579 W/cm^2^, and 785 nm, respectively. The liquid was then substituted by PBS containing 10% CCK-8. After incubation at 37 °C, 1% O_2,_ and 5% CO_2_ for 2 h, a Microplate Reader (ELX800, Bio Tek instruments, Inc., Highland Park, IL, USA) was used to measure the absorbance at 450 nm. The cell morphologies were recorded utilizing an CYTATION/5 imaging reader (Bio Tek instruments, Inc., Winooski, VT, USA).

### 2.12. Intracellular ROS Detection

DU145 prostate cancer cells or PC-3 prostate cancer cells were seeded into each well of the 96-well plates at a density of 1.2 × 10^4^ cells well^−1^ and incubated at 37 °C and 5% CO_2_ for 24 h. Afterwards, the complete medium that was used to incubate the DU145 cells was displaced by CCIPNs or CINs that were diluted with MEM to 0.075 µg mL^−1^ (IR780 concentration), and the complete medium that was used to incubate PC-3 cells was displaced by CCIPNs or CINs that were diluted with DMEM to 0.15 µg mL^−1^ (IR780 concentration). In addition, MEM or DMEM was used to treat the cells and served as a control. After incubation at 37 °C and 5% CO_2_ for 3 h, the liquids were replaced with MEM (for DU145 cells) or DMEM (for PC-3 cells). Subsequently, the cells were treated with or without laser irradiation. The laser was generated by a laser device (MDL-Ⅲ-785 nm-2.5 W-CJ11170, New Industries Optoelectronics Tech. Co., Ltd., Changchun, China). The distance between the laser device and the plate, the optical power density, the wavelength of the laser, and the irradiation time were 20 cm, 0.579 W/cm^2^, 785 nm, and 5 min, respectively. Afterwards, the cells were incubated at 37 °C for 3 h. The medium was then replaced with 10 μmol L^−1^ DCFH-DA (ROS probe), and the cells were incubated at 37 °C for 20 min. After this, serum-free medium was used to wash the cells three times, and a CYTATION/5 imaging reader was used to determine the intensities of fluorescence generated by DCF (Ex/Em = 488/525 nm).

### 2.13. Statistical Analysis

All the data are reported as the mean value ± standard deviation (SD). Statistical analysis was conducted using the SPSS software (edition 16.0, SPSS, Inc., Chicago, IL, USA). After Levene’s test for equality of variances, one-way analysis of variance (ANOVA) analysis was performed. Afterwards, Bonferroni’s multiple comparison test was utilized to compare the mean values. A value of *p* < 0.05 was considered significant.

## 3. Results and Discussion

### 3.1. Selection of Perfluorocarbon Type for CCIPN

**Figure 1:**
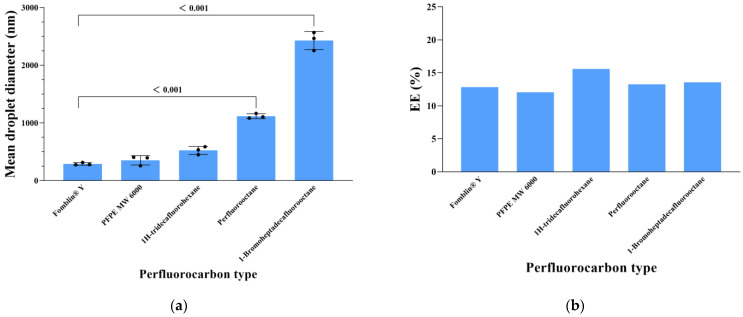
The influence of perfluorocarbon type on the (**a**) mean droplet diameter and (**b**) IR780 EE of emulsions. Values are expressed as mean ± SD (*n* = 3). Each dot represents an independent experiment. The p values vs. indicated groups are presented.

The mean droplet diameters of emulsions prepared by employing various types of perfluorocarbons are shown in Figure 1a. Among the perfluorocarbon types tested, Fomblin^®^ Y (MW 1800) generated the smallest droplets (290.34 ± 21.93 nm), while other types of perfluorocarbons such as perfluorooctane (MW 438.06) and 1-bromoheptadecafluorooctane (MW 498.96) produced large droplets. Meanwhile, the IR780 EE of the droplets produced by Fomblin^®^ Y was acceptable (Figure 1b). In this context, Fomblin^®^ Y was chosen as the optimal perfluorocarbon for CCIPN preparation and used in the following experiments.

### 3.2. Determination of Added CDDO-Me Amount and CAT Distribution Ratio for CCIPN

#### 3.2.1. Effect of Added CDDO-Me Amount on Droplet Size and IR780 EE

**Figure 2:**
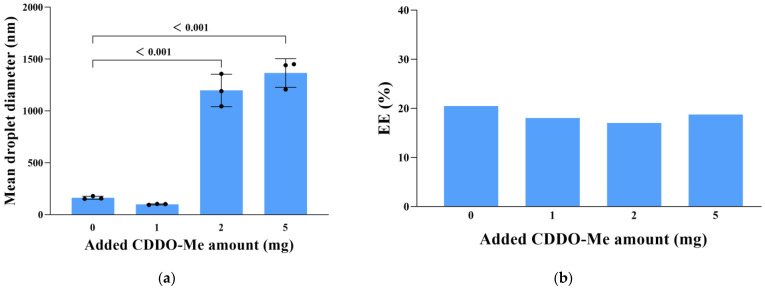
The impact of added CDDO-Me amount on the (**a**) droplet size and (**b**) IR780 EE of emulsions. Values are expressed as mean ± SD (*n* = 3). Each dot represents an independent experiment. The *p* values vs. indicated groups are presented.

As seen from Figure 2a, the droplet size of the emulsion was strongly affected by the amount of CDDO-Me added in the beginning of the preparation process. Small droplets (d < 100 nm) were acquired in the emulsion prepared by adding 1 mg CDDO-Me, while larger droplets were acquired at higher CDDO-Me amounts. In our research, changing the added CDDO-Me amount from 1 mg to 2 mg caused a sharp increase in droplet size. The reason for this phenomenon might be as follows. Usually, a nanoemulsion system has a limited capacity to encapsulate the specific drug. Herein, our nanoemulsion system should have a maximum capacity for loading CDDO-Me. When the fed CDDO-Me amount was 1 mg, our nanoemulsion system might had approached its maximum encapsulating capability. Beyond the maximum capacity, emulsion system ingredients could no longer self-assemble into fine nano- or submicron- sized structures below 1000 nm [27], which was the case in the 2 mg CDDO-Me group. A sharp increase in droplet diameter was also observed in another research work using a nanoemulsion system to deliver vitamin E, as the hydrophobic small molecule payload (vitamin E) feeding amount increased [28]. In this article, the 1 mg added CDDO-Me amount did not induce a significant increase in the droplet size. Meanwhile, the 1 mg added CDDO-Me amount presented an acceptable IR780 EE (Figure 2b). Accordingly, 1 mg CDDO-Me was selected as the optimal input amount and utilized in the succeeding experiments. 

#### 3.2.2. Effect of CAT Distribution on Droplet Size and IR780 EE

**Figure 3:**
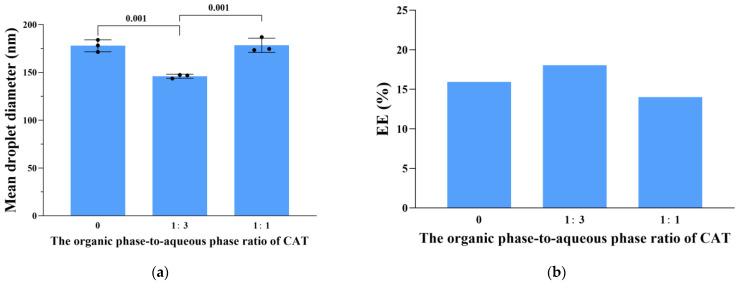
The effect of CAT distribution on the (**a**) droplet size and (**b**) IR780 EE of emulsions. Values are expressed as mean ± SD (*n* = 3). Each dot represents an independent experiment. The *p* values vs. indicated groups are presented.

To evaluate the influence of the CAT distribution among the organic phase and aqueous phase on the droplet size and IR780 EE, the organic-phase-to-aqueous-phase ratio of CAT was set as 0, 1:3, or 1:1. As seen from Figure 3a, the emulsion prepared at a ratio of 1:3 (i.e., ¼ CAT was blended with other ingredients of the organic phase at the start of the preparation process, and ¾ CAT was dispersed in the aqueous phase) had the smallest droplet diameter. Meanwhile, the emulsion produced at a ratio of 1:3 presented a satisfactory IR780 EE (Figure 3b). Therefore, the ratio of 1:3 (i.e., ¼ catalase in organic phase) was considered optimal for CCIPN preparation and was used in subsequent experiments.

### 3.3. Orthogonal Optimization for Preparation of CCIPN Using S-PIC-S Method

The orthogonal experimental design, also known as the factorial design or full factorial design, is a statistical design approach that allows researchers to study the combined effects of multiple variables on an outcome of interest. In an orthogonal design, all possible combinations of the levels of the variables being studied are included in the design, and the effects of the variables are examined independently of one another. This allows researchers to identify the specific contributions of each variable to the outcome and to determine how the variables interact with each other [29].

#### 3.3.1. Orthogonal Experiment and Calculation Results

In order to acquire nanoemulsion droplets with satisfactory indexes (i.e., small diameter and high IR780 EE), the orthogonal experiment was applied. We aimed to search for the optimal level combination of four factors, including the total CAT concentration, ultrasonic power, ultrasonic time, and stirring temperature during the first 10 min (Table 1). According to the characteristic of the orthogonal design, the highest or lowest K value indicates the optimal level of a factor. Therefore, the optimal level of each factor can be determined from calculations based on the orthogonal experiment results. The combination of optimal levels constitutes the soundest plan that we aimed to obtain. In addition, the extent of influence that each factor has on the index (mean droplet diameter or IR780 EE) can be judged according to the R value. The larger R is, the greater influence the factor has on the index.

When the mean droplet diameter was used as an index, the orthogonal experiment and calculation results were as presented in Table 2. The K values indicate that the smallest droplets can be acquired when the preparation condition is A1B2C3D1 (Scheme α). The content of Scheme α is as follows: the total CAT concentration is 2 mg mL^−1^, the ultrasonic power is 60%, the ultrasonic time is 90 s, and the stirring temperature during the first 10 min is 25 °C. Based on the R values, the order of the extent of influence is as follows: ultrasonic time > total CAT concentration > ultrasonic power > stirring temperature during the first 10 min.

When IR780 EE was used as an index, the experimental and calculational results were as shown in Table 3. The K values indicate that when the preparation condition is A1B3C3D1 (Scheme β), the highest IR780 EE can be obtained. Scheme β has the following characteristics: the CAT concentration is 2 mg mL^−1^, the ultrasonic power is 70%, the ultrasonic time is 90 s, and the stirring temperature during the first 10 min is 25 °C. The R values imply that the order of the extent of influence is as follows: ultrasonic time > ultrasonic power > total CAT concentration > stirring temperature during the first 10 min.

#### 3.3.2. Validation of Orthogonal Calculation

The preparation was performed according to Scheme β (A1B3C3D1) or Scheme α (A1B2C3D1). As can be seen from Table 4 and Table 5, the emulsion prepared as per Scheme β had a smaller droplet diameter and higher encapsulation efficiency in comparison with that prepared as per Scheme α. Therefore, Scheme β was determined as the optimal experimental condition. Scheme β has the following characteristics: the total CAT concentration is 2 mg mL^−1^, the ultrasonic power is 70%, the ultrasonic time is 90 s, and the stirring temperature during the first 10 min is 25 °C.

### 3.4. Physicochemical Properties of the Optimized CCIPN

**Figure 4:**
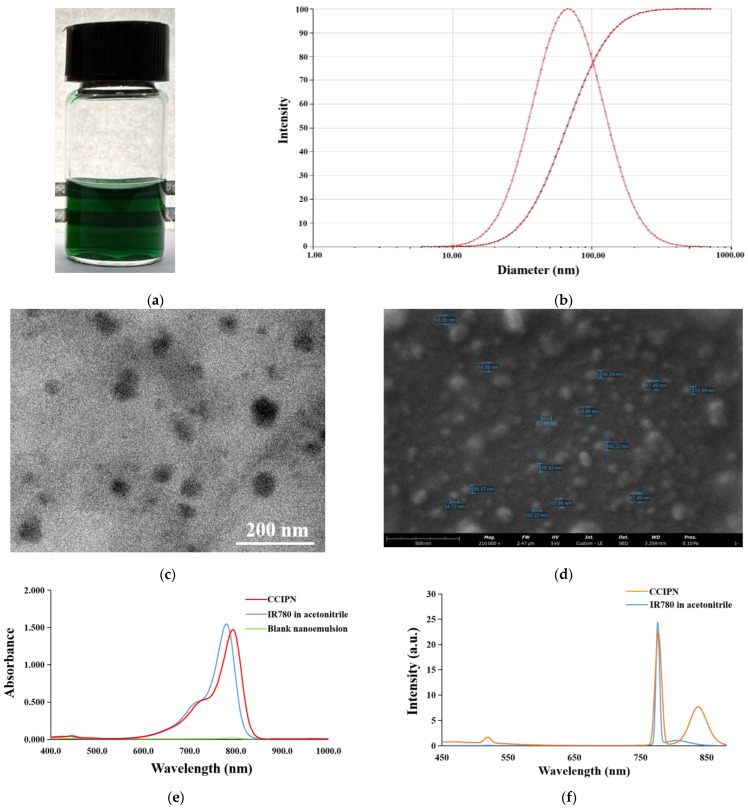
Physicochemical properties of CCIPN. (**a**) The appearance of CCIPN; (**b**) The droplet size distribution of CCIPN; (**c**) Transmission electron microscopy image of CCIPN. Water was used as the aqueous phase to avoid the influence of PBS on the photograph; (**d**) Scanning electron microscopy image of CCIPN. Water was used as the aqueous phase to avoid the influence of PBS on the photograph; (**e**) Absorption spectra of CCIPN, IR780 in acetonitrile, and blank nanoemulsion; (**f**) Fluorescence spectra of CCIPN and IR780 in acetonitrile.

The biomedical application of the near-infrared (NIR) photosensitizer IR780 is impeded by its low aqueous solubility and poor chemical stability [26]. CCIPN enhanced its aqueous solubility through nanoencapsulation. IR780 is innately water-immiscible. However, CCIPN showed a clear and homogeneous appearance, indicating the satisfactory enhancement of the aqueous solubility of IR780 (Figure 4a). Meanwhile, the transparent nature of the appearance suggested that the droplet size was below 100 nm. As seen from Figure 4b, the DLS measurement results indicated that the mean droplet diameter of CCIPN was 65.23 ± 7.39 nm, which was consistent with the visual appearance. The CCIPN droplet diameter is notably smaller than the mean droplet diameters recently published for IR780-P/W NE (205.81 ± 2.25 nm) and FA-IR780 (291.20 ± 82.70 nm) [26,30]. The small droplet size may facilitate the accumulation of CCIPN at the tumor location and improve the therapeutic effects. PDI values describe the narrowness of the droplet size distribution, with a small value indicating a narrow distribution. The PDI value of the optimized CCIPN was 0.355 ± 0.034 (Figure 4b), demonstrating a narrow distribution, which was vital in avoiding droplet growth induced by Ostwald ripening [31]. The SEM and TEM results revealed the spherical morphology of CCIPN droplets (Figure 4c,d). The droplet diameters acquired by the SEM and TEM images agreed with the DLS results.

Figure 4e,f display the UV–visible light absorption spectrum and fluorescence emission spectrum of CCIPN, respectively. As shown in Figure 4e, the blank nanoemulsion (including no IR780) showed no absorption throughout the 400–1000 nm range, whereas CCIPN displayed a prominent absorption peak similar to the peak of IR780 in the acetonitrile solution. This demonstrated that IR780 was effectively encapsulated by the nanoemulsion carrier. The fluorescence emission spectra shown in Figure 4f confirm the conclusion obtained from the UV–visible light absorption spectra. CCIPN showed an identical characteristic peak to IR780 in acetonitrile, demonstrating effective IR780 encapsulation.

### 3.5. Physical and Chemical Stability of CCIPN in Physiological Conditions

**Figure 5:**
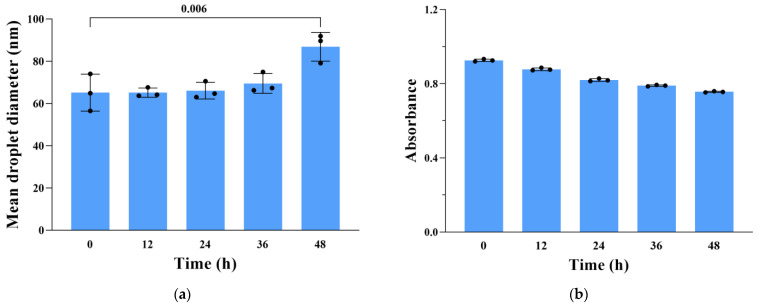
(**a**) Change in CCIPN droplet diameter in pH 7.4 PBS during storage for 48 h at 37 ± 0.5 °C. (**b**) Change in CCIPN absorbance in pH 7.4 PBS during storage for 48 h at 37 ± 0.5 °C. Values are expressed as mean ± SD (*n* = 3). Each dot represents an independent experiment. The *p* values vs. indicated groups are presented.

To investigate the stability of CCIPN in the physiological environment, CCIPN in pH 7.4 PBS was stored for 48 h at 37 °C. During the storage period, the mean droplet diameter was measured by DLS equipment, and the UV–visible spectrophotometer was utilized to determine the absorbance. The absorbance was recorded to detect possible sediments that could not be monitored by appearance observation or DLS measurements. During the 48 h storage period, although slightly increased (Figure 5a), the mean droplet diameter was still less than 100 nm (Figure 5a), which might help to maintain the strength on account of the nano size of CCIPN in biomedical applications. Moreover, the results of absorbance (Figure 5b) showed that the IR780 remained up to 81.7% after 48 h. The small decrease in IR780 content was likely caused by the IR780’s degradation rather than sedimentation after storage for 48 h at 37 °C, because IR780 is easily degraded when the temperature is increased. In brief, the results of the absorbance tests demonstrated that the location of IR780 was within the emulsion system rather than sedimentation, which suggested the reliability of the DLS results. In conclusion, the results above indicate that the nanoemulsion’s encapsulation might promote the biomedical application of IR780 by extending the circulation time of IR780.

### 3.6. Physical and Chemical Stability of CCIPN during Storage at 4 °C

**Figure 6:**
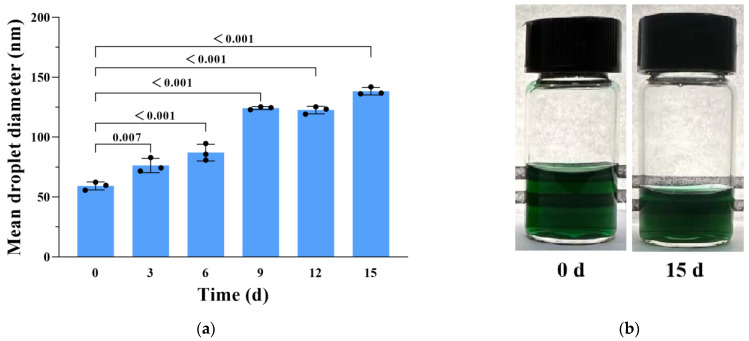
(**a**) The change in droplet diameter of CCIPNs during storage at 4 °C for 15 days. (**b**) The appearance of CCIPNs preserved at 4 ◦C and stored for 0 or 15 days. Values are expressed as mean ± SD (*n* = 3). Each dot represents an independent experiment. The p values vs. indicated groups are presented.

The DLS equipment was used to investigate the physical stability of CCIPN during storage at 4 °C. Although it rose to 138.30 ± 3.13 nm (the 15th day), the mean droplet diameter was still below 150 nm (Figure 6a). Meanwhile, no creaming or precipitation was detected, and the CCIPN maintained a transparent and homogeneous appearance over the 15-day duration (Figure 6b). This is beneficial for the transport and application of CCIPN. It is possible that as a type of protein, the catalase in the CCIPN underwent a conformational change as time passed in the aqueous environment, leading to the elevation of the droplet size.

**Figure 7:**
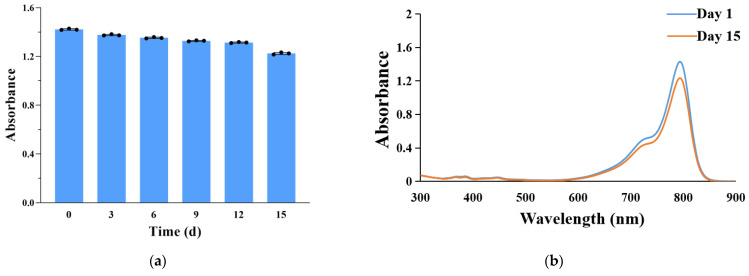
(**a**) The change in absorbance of CCIPNs during storage at 4 °C for 15 days. (**b**) The change in ultraviolet–visible light absorption spectrum of CCIPNs during storage at 4 °C for 15 days. Values are expressed as mean ± SD (*n* = 3). Each dot represents an independent experiment.

The changes in the absorbance at 793 nm and the UV–visible light absorption spectrum were recorded to evaluate the chemical stability of CCIPN during 15-day storage at 4 °C. As seen from Figure 7a, after storage at 4 °C for 15 days, the IR780 retention rate was above 86%. The absorption spectrum of CCIPN presented a trivial alteration before and after 15-day storage at 4 °C (Figure 7b). This indicated that the packing of IR780 in nanoemulsion formulations resulted in high chemical stability. The chemical stability could be ascribed to the interfacial protective layer created by the emulsifier molecules.

### 3.7. Cytocompatibility Assays

**Figure 8:**
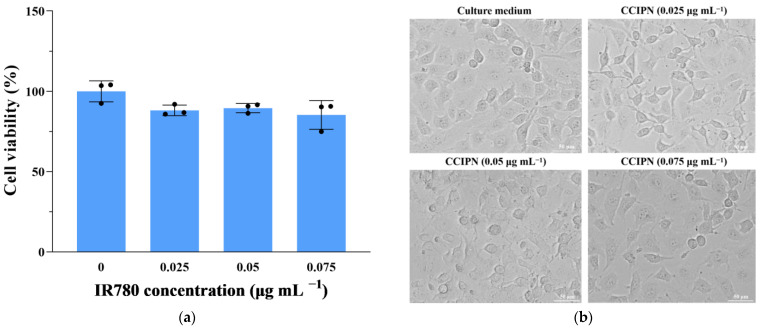
(**a**) Viability of HUVECs after incubation with various concentrations of CCIPNs (IR780 concentration 0, 0.025, 0.05 and 0.075 μg mL^−1^) for 24 h in the dark. (**b**) Morphologies of HUVECs after incubation with various concentrations of CCIPNs (IR780 concentration 0, 0.025, 0.05 and 0.075 μg mL^−1^) for 24 h in the dark. Values are expressed as mean ± SD (*n* = 3). Each dot represents an independent experiment.

To evaluate the CCIPN’s compatibility with normal cells, we detected the viability and morphologies of HUVEC (a non-cancerous cell line) cells incubated with various concentrations of CCIPNs for 24 h in the dark at 37 °C and 5% CO_2_. As is shown in Figure 8a, no significant change in cell viability was observed within our concentration range (0–0.075 μg mL^−1^). As presented in Figure 8b, consistent with the cell viability results, the cells at every concentration presented no detectable morphological alteration in comparison with the culture medium group. The cells retained their normal elongated or fusiform shapes similar to the morphologies of the cells incubated with only the culture medium. These results concerning cell viability and morphology indicate the satisfactory cytocompatibility of CCIPN.

**Figure 9:**
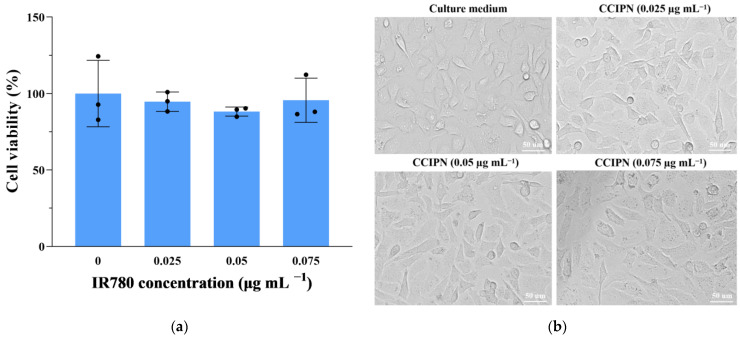
(**a**) Viability of DU145 prostate cancer cells after incubation with various concentrations of CCIPNs (IR780 concentration 0, 0.025, 0.05, and 0.075 μg mL^−1^) for 24 h in the dark. (**b**) Morphologies of DU145 prostate cancer cells after incubation with various concentrations of CCIPNs (IR780 concentration 0, 0.025, 0.05 and 0.075 μg mL^−1^) for 24 h in the dark. Values are expressed as mean ± SD (*n* = 3). Each dot represents an independent experiment.

PDT uses light as a switch to control the cytotoxicity of a photosensitizer, killing cancer cells selectively. In this context, excellent cytocompatibility in the absence of light is essential for a PDT agent [32]. Therefore, the cytocompatibility of CCIPN with DU145 prostate cancer cells under dark conditions was studied. After the cells were incubated with CCIPNs at diverse concentrations (0–0.075 μg mL^−1^) in the dark for 24 h at 37 °C and 5% CO_2_, almost no cell viability variation could be detected (Figure 9a). Moreover, in agreement with the cell viability results, the cells at each concentration presented no detectable morphological change in comparison with the control group (Figure 9b). The cells retained their normal elongated or fusiform shapes, similar to the morphologies of the control group cells. These results demonstrate the satisfactory cytocompatibility of CCIPN in darkness, i.e., negligible “dark toxicity” [33].

### 3.8. Phototoxicity Assays

**Figure 10:**
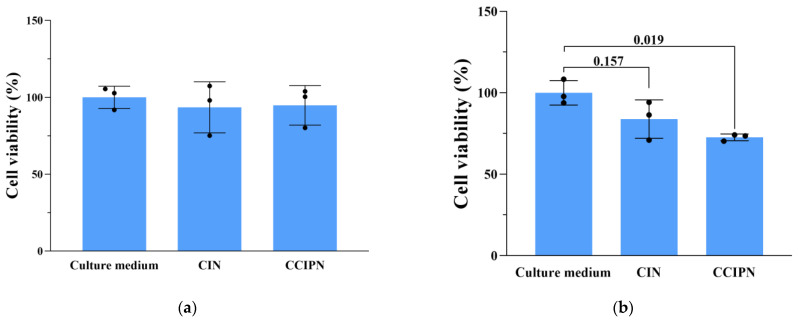
Investigation of the phototoxicity of CCIPN. (**a**) Viability of DU145 cells treated with culture medium, CCIPN at 0.075 µg mL^−1^, or CIN at 0.075 µg mL^−1^ under no irradiation. (**b**) Viability of DU145 cells treated with culture medium, CCIPN at 0.075 µg mL^−1^, or CIN at 0.075 µg mL^−1^ under irradiation. Values are expressed as mean ± SD (*n* = 3). Each dot represents an independent experiment. The *p* value vs. indicated group is presented.

**Figure 11:**
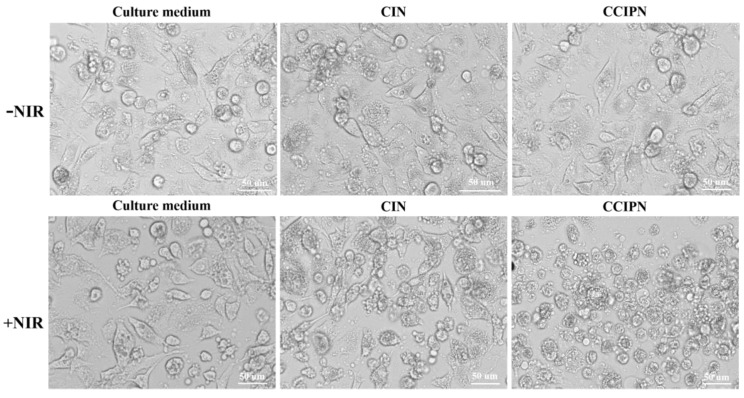
DU145 cell morphologies under different treatment conditions.

In order to examine the anticancer effect in vitro, cell viability measurements were performed using the CCK-8 method, and cell morphology inspection was conducted utilizing a CYTATION/5 imaging reader. Under hyperoxic condition (~21% O_2_), DU145 prostate cancer cells were incubated with CCIPN or CIN having the same IR780 concentration as the CCIPN, or serum-free medium, and then treated with or without 785 nm laser irradiation. As seen from Figure 10a, in the absence of light, the cells incubated with culture medium, CIN, or CCIPN presented no variation in cell viability. This indicated that no tumor cell killing could be achieved without light illumination. As seen from Figure 10b, a slightly (non-significantly) greater reduction in cell viability was observed in the CCIPN + NIR group than in the CIN + NIR group, demonstrating the higher efficiency of the CCIPN in destroying tumor cells upon light stimulation. Furthermore, the cell morphologies of the treatment groups are shown in Figure 11. In agreement with the cell viability results, the cells in the CCIPN group or CIN group without illumination showed little morphological variation in comparison with the control group. The DU145 cells maintained their normal elongated or fusiform shapes (Figure 11—NIR groups). In the CIN + NIR group, the majority of cells maintained their normal fusiform or elongated shapes similar to the control group. In sharp contrast, nearly all the cells in the CCIPN + NIR group contracted to spherical shapes, indicating severe damage to the tumor cells (Figure 11, + NIR groups). The high solubility of Fomblin^®^ Y compounds for oxygen and the generation of oxygen by catalase should be critical reasons for the high DU145 cell-killing efficiency. In summary, these results suggest that CCIPN could enhance the phototherapeutic influence on tumor cells under NIR light activation because of the increased oxygen supply. For prostate cancer, severe hypoxia has been reported to be closely related to disease aggressiveness [34], and it constitutes an important obstacle for immunotherapy [35]. The superior cytotoxicity to DU145 prostate cancer cells promoted by oxygen supplementation makes CCIPN a candidate to enhance prostate cancer immunotherapy via combating hypoxia. Meanwhile, CDDO-Me has been reported to vigorously inhibit the proliferation and promote the apoptosis of diverse types of cancer cell lines [36,37,38]. It was also stated that CDDO-Me could regulate the immunosuppressive tumor microenvironment and coordinate with vaccine therapy [24]. Therefore, if CCIPN is combined with immunotherapies, it might improve the therapeutic effect by relieving hypoxia through catalase & perfluoropolyether and remodeling the immunosuppressive tumor microenvironment through CDDO-Me.

We further studied the phototoxicity of CCIPN on a non-cancerous cell line, HUVEC cells under hyperoxic condition (~21% O_2_). To examine the influence of CCIPN on HUVECs, the CCK-8 method was used to measure cell viability, and a CYTATION/5 imaging reader was applied to record the cells’ morphologies. HUVECs were incubated with CCIPN, CIN having the same IR780 concentration as the CCIPN, or serum-free medium, and then treated with or without 785 nm laser irradiation. 

**Figure 12:**
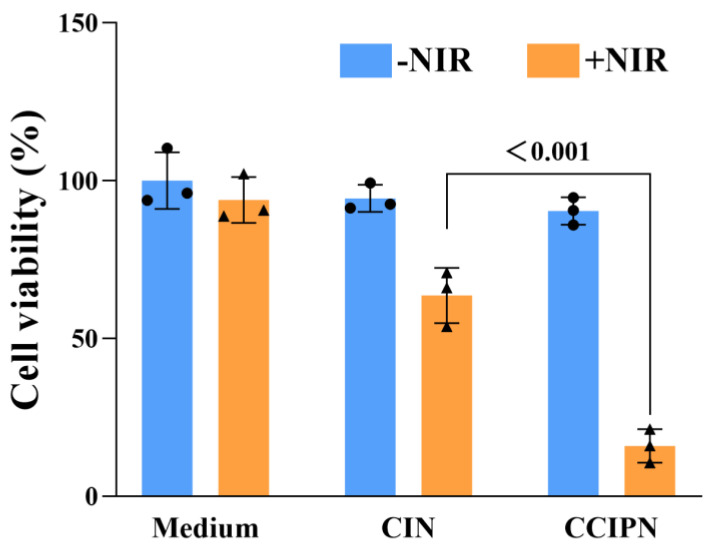
Investigation of the phototoxicity of CCIPN. Viability of HUVECs treated with culture medium, CCIPN at 0.075 µg mL^−1^ or CIN at 0.075 µg mL^-1^ under irradiation or no irradiation. Values are expressed as mean ± SD (*n* = 3). Each dot represents an independent experiment. The *p* value vs. indicated group is presented.

**Figure 13:**
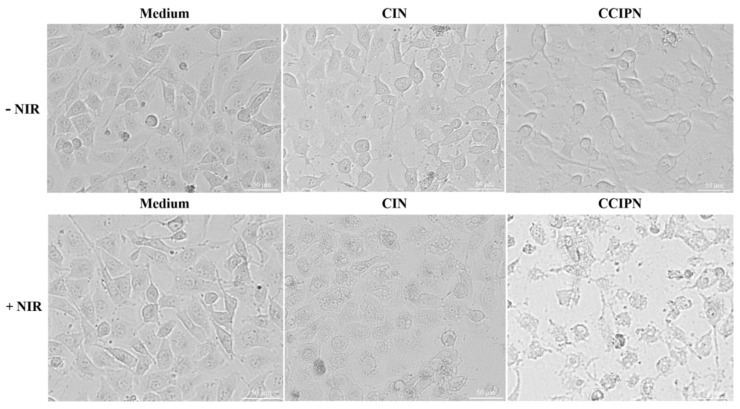
HUVECs morphologies under different treatment conditions.

As presented in Figure 12, without light irradiation, the cells incubated with the culture medium, CIN or CCIPN, showed little alteration in cell viability. This is in agreement with the abovementioned cytocompatibility assay results and indicates that no cell damage could occur without light stimulation. In addition, the cell viability of the Medium + NIR group was similar to that in the Medium—NIR group, indicating that the light irradiation condition (optical power density, irradiation time, etc.) itself could not elicit cytotoxicity. Upon light irradiation, the cells cultured with CCIPN showed a greater decrease in cell viability compared with the cells incubated with CIN. Furthermore, the cell morphology results agree with the cell viability data (Figure 13). The cellular shapes of the – NIR groups and Medium + NIR group were similar, being elongated, suggesting that the cells were in a normal state. The CCIPN + NIR group presented a greater number of round cells in comparison with the CIN + NIR group, which indicated more severe damage to cells. The light-responsive property of CCIPN here is consistent with the abovementioned phototoxicity assay results on the DU145 prostate cancer cells. The results here suggest that the CCIPN could enter both cancerous and normal cells, and cause side effects if the normal cells were irradiated accidentally or due to other reasons. However, this side effect may be avoided by strictly confining the irradiation area to only tumor sites. Future modification to CCIPN, such as conjugating tumor-targeting moieties (e.g., folic acid, or ligands targeting CD44), might realize a “double targeting” (tumor-targeting moiety and light) effect, and benefit antitumor treatment outcomes [39,40].

**Figure 14:**
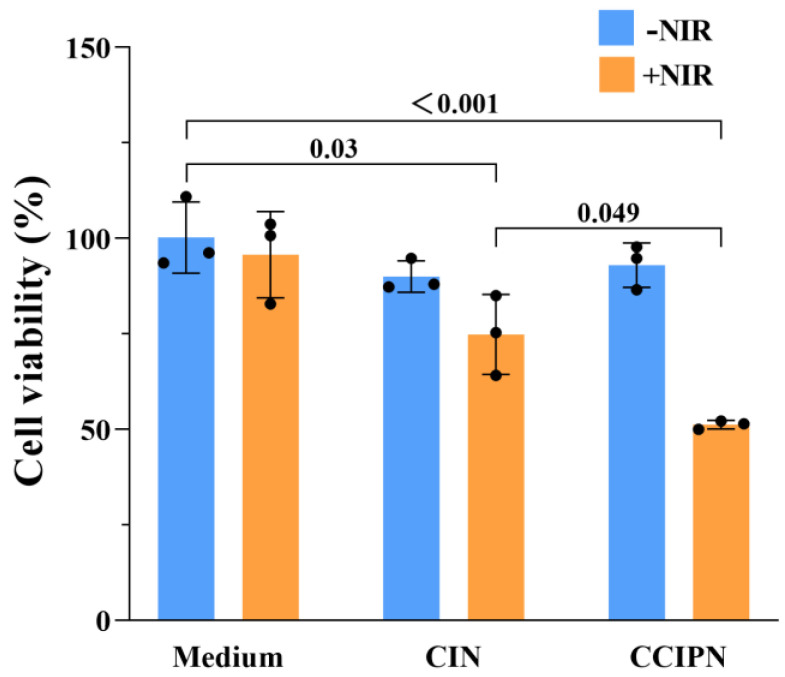
Investigation of the phototoxicity of CCIPN under hypoxic condition. Viability of PC-3 cells treated with culture medium, CCIPN at 0.075 µg mL^−1^, or CIN at 0.075 µg mL^−1^ under irradiation or no irradiation. Values are expressed as mean ± SD (*n* = 3). Each dot represents an independent experiment. The *p* values vs. indicated groups are presented.

**Figure 15:**
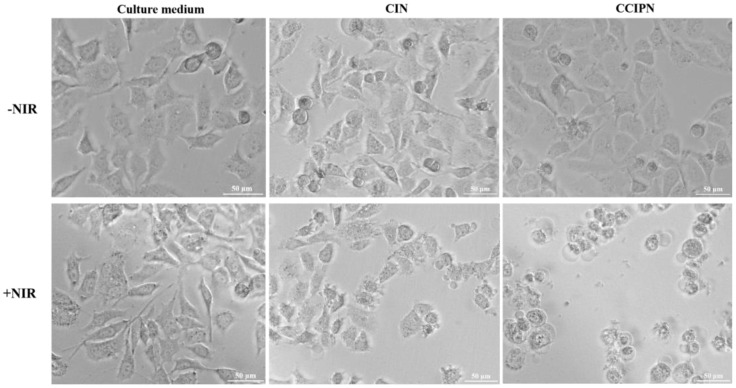
PC-3 cell morphologies after receiving different treatments in hypoxic condition.

We further studied the phototoxicity of CCIPN in comparison with CIN under hypoxic condition. The experimental O_2_ concentration was set as 1% in order to simulate the hypoxic tumor microenvironment [41,42]. As presented in Figure 14, the CCIPN + NIR treatment induced a lower cell viability compared with CIN + NIR treatment, suggesting the superior ability of CCIPN to destruct cells under light irradiation in hypoxic condition. It is noteworthy that neither Medium + NIR group nor − NIR groups (CCIPN − NIR group and CIN − NIR group) presented significant cell viability change in comparison with the Medium − NIR group, indicating that neither irradiation alone nor drug incubation alone could cause cell death. This demonstrated that the cell viability reductions seen in the CCIPN + NIR group and CIN + NIR group were caused by the combinatory effect of light and drug. In other words, the results here suggest that CCIPN could induce stronger photodynamic effect than CIN. This might be ascribed to the oxygen-supplying ability of catalase and Fomblin^®^ Y in the CCIPN. As shown in Figure 15, the cell morphology detection results agree with the cell viability data. The CIN + NIR group presented elongated morphology similar to the Culture medium group, while the CCIPN + NIR group exhibited a contracted spherical shape. Neither drug alone nor irradiation alone elicited obvious cell morphology change. In this article, most of the in vitro cellular experiments were performed in hyperoxic condition, and this is a strong limitation of the present study. More in vitro experiments under hypoxic condition and in vivo tests remain to be performed to provide information on the potential of CCIPN to work in the hypoxic tumor microenvironment.

### 3.9. Intracellular ROS Production by CCIPN

**Figure 16:**
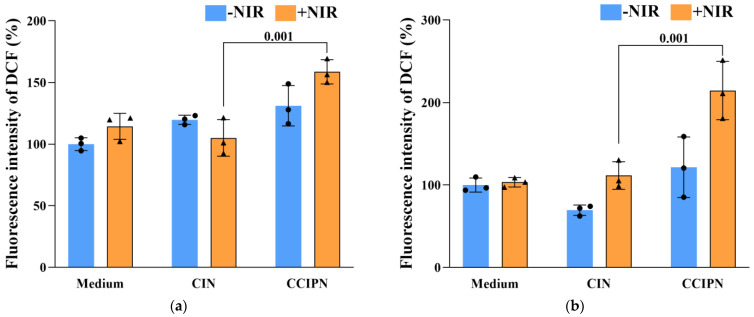
(**a**) Generation of intracellular reactive oxygen species indicated by DCF in DU145 cells treated with culture medium, CCIPN at 0.075 µg mL^−1^ or CIN at 0.075 µg mL^−1^ with/without NIR light irradiation. (**b**) Generation of intracellular reactive oxygen species indicated by DCF in PC3 cells treated with culture medium, CCIPN at 0.15 µg mL^−1^ or CIN at 0.15 µg mL^−1^ with/without NIR light irradiation. Values are expressed as mean ± SD (*n* = 3). Each dot represents an independent experiment. The *p* values vs. indicated groups are presented.

After determining the ability of CCIPN to kill cells in response to light stimulation, we further explored the mechanism underlying the phototoxicity in terms of intracellular ROS production. The intracellular ROS generated by CCIPN and CIN at the same IR780 concentration with or without NIR irradiation was quantified utilizing a fluorogenic probe DCFH-DA, with serum-free medium serving as a control. After 785 nm laser irradiation, the DU145 prostate cancer cells incubated with CCIPN showed clearly higher fluorescence intensity compared with those incubated with CIN (1.51-fold, Figure 16a). This demonstrated that more ROS was generated by the CCIPN under laser irradiation. Consistently, tests on another prostate cancer cell line, PC-3, showed that the fluorescence intensity of the CCIPN + NIR group was clearly higher than that of the CIN + NIR group (1.93 fold, Figure 16b), which indicated the higher efficiency of CCIPN in generating cytotoxic ROS upon light stimulation in comparison with CIN. The results here are in agreement with other research using catalase [43] or perfluorocarbon [44] to increase the PDT efficacy. In summary, the results here imply that CCIPN may boost the cytotoxic ROS production through oxygen supplementation, leading to enhanced phototoxicity.

## 4. Conclusions

In this research, a hydrogen peroxide-responsive and oxygen-gathering nanoemulsion system, CCIPN, was rationally designed to address hypoxia in PDT. With perfluoropolyether and catalase converged in one nanoplatform CCIPN, the oxygen generated by the decomposition of tumor-overproduced hydrogen peroxide via catalase could be reserved by perfluoropolyether for PDT. CCIPN represents a hypoxic tumor PDT strategy integrating the high tumor specificity of the oxygen generation approach and the high efficiency of the oxygen delivery tactic. CCIPN was produced using the S-PIC-S method, which we developed from our previous SPIC method, and the preparation process was optimized using orthogonal tests. The prepared CCIPN contained spherical droplets below 100 nm in size, with a considerable amount of catalase loaded. It showed high cytocompatibility in the dark and enhanced ROS generation and cytotoxicity under light irradiation, which indicated its feasibility in supplementing oxygen and boosting PDT efficiency. Moreover, with the ability to combat hypoxia (which is also an obstacle in tumor immunotherapy) and the potential to regulate the immunosuppressive tumor microenvironment (endowed by the loaded CDDO-Me), CCIPN may be a promising agent to activate the immune system and be synergized with immunotherapy in the future. 

## Figures and Tables

**Table 1:** Factors and levels in the design of the orthogonal experiment.

Level	Factor			
	A	B	C	D
1	2	50	30	25
2	4	60	60	30
3	6	70	90	35

A (Total CAT concentration, mg mL^−1)^, B (Ultrasonic power, %), C (Ultrasonic time, s), D (Stirring temperature during the first 10 min, °C).

**Table 2:** Orthogonal experiment design and results concerning mean droplet diameter.

Experiment Number	Factor				Mean Droplet Diameter (nm)
	A	B	C	D	
1	2	50	30	25	428.57
2	2	60	60	30	433.00
3	2	70	90	35	312.76
4	4	50	60	35	825.13
5	4	60	90	25	326.12
6	4	70	30	30	501.58
7	6	50	90	30	449.51
8	6	60	30	35	546.12
9	6	70	60	25	570.00
K1	1174.33	1703.21	1476.27	1324.69	
K2	1652.83	1305.24	1828.13	1384.09	
K3	1565.63	1384.34	1088.39	1684.01	
R	159.50	132.66	246.58	119.77	

A (Total CAT concentration, mg mL^−1)^, B (Ultrasonic power, %), C (Ultrasonic time, s), D (Stirring temperature during the first 10 min, °C). Ki represents the sum of the test results with level number i within any column. R stands for range, i.e., the averaged result of maximum Ki average minus minimum Ki. The smallest K value indicates the optimal level of the factor. The larger the R-value is, the greater influence the factor has on the index.

**Table 3:** Orthogonal experiment design and results concerning encapsulation efficiency.

Experiment Number	Factor				Encapsulation Efficiency (%)
	A	B	C	D	
1	2	50	30	25	20.95
2	2	60	60	30	22.51
3	2	70	90	35	32.14
4	4	50	60	35	22.13
5	4	60	90	25	25.57
6	4	70	30	30	20.77
7	6	50	90	30	23.44
8	6	60	30	35	18.61
9	6	70	60	25	26.75
K1	75.60	66.52	60.33	73.27	
K2	68.47	66.69	71.39	66.72	
K3	68.80	79.66	81.15	72.88	
R	2.38	4.38	6.94	2.18	

A (Total CAT concentration, mg mL^−1)^, B (Ultrasonic power, %), C (Ultrasonic time, s), D (Stirring temperature during the first 10 min, °C). Ki represents the sum of the test results with level number i within any column. R stands for range, i.e., the averaged result of maximum Ki average minus minimum Ki. The highest K value indicates the optimal level of the factor. The larger the R-value is, the greater influence the factor has on the index.

**Table 4:** Experimental results on mean droplet diameters of Scheme α and Scheme β.

Scheme	Factor				Mean Droplet Diameter (nm, ± SD)
α	A1	B2	C3	D1	73.80 ± 12.74
β	A1	B3	C3	D1	57.14 ± 9.49

A1 (Total CAT concentration is 2 mg mL^−1^), B2 (Ultrasonic power is 60%), B3 (Ultrasonic power is 70%), C3 (Ultrasonic time is 90 s), D1 (Stirring temperature during the first 10 min is 25 °C).

**Table 5:** Experimental results on IR780 encapsulation efficiencies of Scheme α and Scheme β.

Scheme	Factor				Encapsulation Efficiency (%)
α	A1	B2	C3	D1	22.84
β	A1	B3	C3	D1	32.36

A1 (Total CAT concentration is 2 mg mL^−1^), B2 (Ultrasonic power is 60%), B3 (Ultrasonic power is 70%), C3 (Ultrasonic time is 90 s), D1 (Stirring temperature during the first 10 min is 25 °C).

## Data Availability

The data presented in this study are available in this article.
